# Shortwave infrared emitting multicolored nanoprobes for biomarker-specific cancer imaging in vivo

**DOI:** 10.1186/s12885-020-07604-8

**Published:** 2020-11-10

**Authors:** Harini Kantamneni, Shravani Barkund, Michael Donzanti, Daniel Martin, Xinyu Zhao, Shuqing He, Richard E. Riman, Mei Chee Tan, Mark C. Pierce, Charles M. Roth, Vidya Ganapathy, Prabhas V. Moghe

**Affiliations:** 1 Department of Chemical & Biochemical Engineering, Rutgers University, 98 Brett Road, Piscataway, NJ 08854 USA; 2 Department of Biomedical Engineering, Rutgers University, 599 Taylor Road, Piscataway, NJ 08854 USA; 3 Engineering Product Development, Singapore University of Technology and Design, 8 Somapah Rd, Singapore, 487372 Singapore; 4 Department of Materials Science and Engineering, Rutgers University, 607 Taylor Road, Piscataway, NJ 08854 USA

**Keywords:** Cancer metastasis, Nanotechnology, Short-wave infrared imaging, Multiplexing, Rare earths

## Abstract

**Background:**

The ability to detect tumor-specific biomarkers in real-time using optical imaging plays a critical role in preclinical studies aimed at evaluating drug safety and treatment response. In this study, we engineered an imaging platform capable of targeting different tumor biomarkers using a multi-colored library of nanoprobes. These probes contain rare-earth elements that emit light in the short-wave infrared (SWIR) wavelength region (900–1700 nm), which exhibits reduced absorption and scattering compared to visible and NIR, and are rendered biocompatible by encapsulation in human serum albumin. The spectrally distinct emissions of the holmium (Ho), erbium (Er), and thulium (Tm) cations that constitute the cores of these nanoprobes make them attractive candidates for optical molecular imaging of multiple disease biomarkers.

**Methods:**

SWIR-emitting rare-earth-doped albumin nanocomposites (ReANCs) were synthesized using controlled coacervation, with visible light-emitting fluorophores additionally incorporated during the crosslinking phase for validation purposes. Specifically, HoANCs, ErANCs, and TmANCs were co-labeled with rhodamine-B, FITC, and Alexa Fluor 647 dyes respectively. These Rh-HoANCs, FITC-ErANCs, and 647-TmANCs were further conjugated with the targeting ligands daidzein, AMD3100, and folic acid respectively. Binding specificities of each nanoprobe to distinct cellular subsets were established by in vitro uptake studies. Quantitative whole-body SWIR imaging of subcutaneous tumor bearing mice was used to validate the in vivo targeting ability of these nanoprobes.

**Results:**

Each of the three ligand-functionalized nanoprobes showed significantly higher uptake in the targeted cell line compared to untargeted probes. Increased accumulation of tumor-specific nanoprobes was also measured relative to untargeted probes in subcutaneous tumor models of breast (4175 and MCF-7) and ovarian cancer (SKOV3). Preferential accumulation of tumor-specific nanoprobes was also observed in tumors overexpressing targeted biomarkers in mice bearing molecularly-distinct bilateral subcutaneous tumors, as evidenced by significantly higher signal intensities on SWIR imaging.

**Conclusions:**

The results from this study show that tumors can be detected in vivo using a set of targeted multispectral SWIR-emitting nanoprobes. Significantly, these nanoprobes enabled imaging of biomarkers in mice bearing bilateral tumors with distinct molecular phenotypes. The findings from this study provide a foundation for optical molecular imaging of heterogeneous tumors and for studying the response of these complex lesions to targeted therapy.

**Supplementary Information:**

The online version contains supplementary material available at 10.1186/s12885-020-07604-8.

## Background

Targeted therapy relies on variability among molecular biomarkers to inform the oncologist on treatment decisions and to predict the success of a chosen regimen [1–6]. Current methods of assessing a tumor’s molecular signature involve biopsy sampling, which accesses only a small subset of the tumor tissue, underestimates molecular phenotypic variability [7, 8], and is impractical for evaluation of temporal changes in tumor properties. These molecular drivers can also serve as imaging biomarkers for non-invasive studies of tumor composition and dynamic behavior [9], with particular relevance in early preclinical studies of drug safety and therapeutic efficacy [10–12]. However, existing imaging modalities such as MRI, CT, and PET/SPECT offer limited options for multi-spectral molecular phenotyping. Optical imaging can potentially fill this gap in preclinical evaluation of targeted therapies, but new probes capable of tracking multiple imaging biomarkers are required.

Here we demonstrate a set of spectrally-distinct, biomarker-specific, optical nanoprobes for detection of varying molecular subtypes in in vivo murine tumor models. These nanoprobes are based on ceramic rare-earth (RE) doped nanoprobes encapsulated in human serum albumin, forming rare-earth albumin nanocomposites (ReANCs) [13, 14]. ReANCs are excited using near-infrared (NIR) light (980 nm) and emit at short-wave infrared (SWIR) wavelengths (900–1700 nm), allowing for superior imaging depth, contrast, and resolution compared to visible fluorophores [13–15]. Both passive and active targeting of ReANCs have been shown to influence nanoprobe biodistribution, provide molecular information on a region of interest, and improve signal-to-background ratio on imaging, allowing for highly sensitive assessment of microscopic lesions [16, 17]. We previously demonstrated the unique capabilities of erbium-doped ReANCs for surveillance of multi-organ metastases using a cocktail of niche-targeted probes in single animals with excellent safety and clearance profiles [18]. Additionally, a number of recent studies have also highlighted the distinct imaging potential of rare-earth based nanoparticles in tumor imaging [19–23]. These nanoprobes have been repeatedly in our studies shown to clear completely subsequent to both intra-peritoneal and intravenous administration and exhibit little to no toxicity following repeated administration [13, 18].

**Fig. 1:**
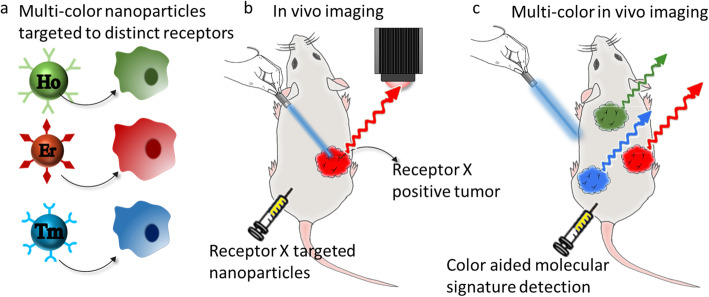
Multi-color nanoprobes for biomarker-specific in vivo imaging. **a** Multi-color nanoprobes were engineered to target biomarkers specific to different breast cancer cell lines. **b** Whole body SWIR imaging was used to assess in vivo localization of targeted nanoprobes evaluated in subcutaneous tumor models. **c** A bilateral tumor model was used to assess the specificity of multi-color nanoprobes to tumors expressing distinct biomarkers

In this study, we used three different rare-earth dopants to generate a set of nanoprobes with distinct emission spectra [24, 25] for multispectral imaging of several tumor biomarkers. We used holmium (Ho) emissions at 1185 nm, erbium (Er) emissions at 1525 nm, and thulium (Tm) emissions at 1475 nm to target cellular subsets with unique cell-surface receptor expression patterns (Fig. 1). Notably, these emissions were all generated by using a single illumination wavelength (980 nm). The nanoprobes were also modified to contain conventional, visible light-emitting fluorophores (rhodamine-B, FITC, and Alexa Fluor 647) for microscopic imaging on commercial platforms. The affinity of each nanoprobe to its targeted biomarker was quantified using flow cytometry. The in vivo targeting ability of each nanoprobe was established by imaging mice bearing subcutaneous tumors expressing one of three biomarkers of relevance in breast cancer: caveolin 1 (CAV1), C-X-C chemokine receptor type 4 (CXCR4), and Folate Receptor alpha (FRα) (Table 1). We conclude by showing that these multispectral nanoprobes enable image-based differentiation of tumors with different molecular phenotypes in individual animals bearing bilateral tumors.

**Table 1:** Summary of targeted biomarkers and their relative expression levels in breast (MCF7, 4175TR) and ovarian (SKOV3) cancer cell lines. Also listed are the corresponding targeting ligands and nanoparticle compositions

	Biomarker		
	*CAV1*	*CXCR4*	*FRα*
*MCF7 expression*	+	+++	++
*4175TR expression*	+++	+	–
*SKOV3 expression*	+++	–	+++
*Targeting ligand*	Daidzein	AMD3100	Folic acid
*Visible / SWIR reporter*	Rhodamine / Ho	FITC / Er	Alexa Fluor 647 / Tm
*Targeted nanoparticle*	Rh-fHoANC	FITC-fErANC	647-fTmANC

## Methods

### Cell lines

MCF7 (ATCC) and 4175TR cells (a kind gift from Dr. Yibin Kang) [26, 27] were cultured in DMEM media supplemented with 10% FBS (Atlanta Biologicals) and 1% penicillin-streptomycin (P/S) (Gibco Inc.). SKOV3 cells (a kind gift from by Dr. Steven K. Libutti) were cultured in McCoy’s media with FBS and P/S supplements.

### Rare-earth nanoprobe synthesis

Holmium, erbium, and thulium doped nanoprobe cores were synthesized via a burst nucleation process, as described previously [13]. Holmium doped rare-earth (Ho-RE) cores were encapsulated in albumin using a controlled coacervation method as described in previous publications [13, 15, 18]. These nanoprobes were further loaded with rhodamine-B during the coacervation process. Briefly, Ho-REs in ethanol (0.5 mg/mL) were sonicated with 2.53% (v/v) of rhodamine-B stock solution (1 mg/mL). Two mL of the rhodamine-infused Ho-RE solution was added at a rate of 1.5 mL/min to 500 μl of 20% (w/v) human serum albumin solution in 10 mM NaCl, with pH adjusted to 8.5, under constant stirring at 700 rpm. Next, 2.34 μl of glutaraldehyde was added to the resulting mixture and the solution allowed to crosslink overnight under constant stirring. Post-encapsulation, the resulting Rh-HoANCs were purified via centrifugation at 20,000 rpm for three cycles of 10 min each. Erbium doped rare-earth (Er-RE) core nanoprobes were encapsulated similarly to the method described above. Er-REs in ethanol (0.2 mg/mL) were sonicated with 2.53% (v/v) of FITC stock solution (1 mg/mL). Two mL of the FITC-infused Er-RE solution was added at a rate of 1.5 mL/min to 500 μl of 20% (w/v) human serum albumin solution in 10 mM NaCl, with pH adjusted to 8.5, under constant stirring at 700 rpm. Subsequently, 2.34 μl of glutaraldehyde was added to the resulting mixture and the solution allowed to crosslink overnight under constant stirring. The FITC-ErANCs were then purified by centrifugation as described above. Thulium doped rare-earth (Tm-RE) core probes were encapsulated as above with the Tm-REs in ethanol at a concentration of 1 mg/mL sonicated with 0.31% (v/v) of Alexa Fluor 647 stock solution (1 mg/mL). The 647-TmANCs were purified by centrifugation as described above.

Dynamic light scattering (DLS) (Malvern Instruments) was used to measure the hydrodynamic diameter and polydispersity index (PDI) of all formulations of ReANCs (**Supplementary Fig.** **1**). The emission spectra of fluorophore-free (HoANCs, ErANCs, TmANCs) and fluorophore-loaded (Rh-HoANCs, FITC-ErANCs, 647-TmANCs) nanoprobes were measured with a Zeiss LSM780 confocal microscope equipped with a Quasar 32-channel spectral detector. Additionally, the relative emission intensities of Rh-HoANCs, FITC-ErANCs, and 647-TmANCs nanoprobes were measured within their respective emission bands in the rhodamine(Ex:510 nm), FITC(Ex:488 nm) and Alexa-647(Ex:647 nm) region was quantified using a fluorescence plate reader.

### Rare-earth nanoprobe functionalization

Rh-HoANCs were conjugated to daidzein via adsorption onto albumin drug binding pockets [15] at daidzein concentrations ranging from 3.93 μM to 393 μM, forming functionalized nanocomposites (Rh-fHoANCs). Higher concentrations of daidzein resulted in the formation of aggregates. Relative cellular uptake by FACS analysis (described below) determined the optimal concentration to be 393 μM, which was used to functionalize Rh-HoANCs for subsequent in vivo experiments. FITC-ErANCs were modified with AMD3100 via adsorption at bulk concentrations ranging from 12.5 nM to 125 μM. A concentration of 1.25 μM was found to be optimal from FACS analysis and was used to fabricate functionalized nanoprobes (FITC-fErANCs) for subsequent in vivo applications. Alexa 647-fTmANCs were synthesized by chemical conjugation of folic acid (FA) using a 1-ethyl-3-(3-dimethylaminopropyl)carbodiimide (EDC) (Thermofisher) crosslinker with a zero-space linker. This was achieved by crosslinking the amine groups present on the nanoprobes with carboxylic acid groups present on the FA ligand. First, 3.5 mL of FA stock solution at a concentration of 10 mg/mL in 0.1 M NaOH was activated by the addition of 7 mg of EDC at a final concentration of 2 mM and incubated in the dark at room temperature for 15 min. The activated FA was added dropwise to 3.5 mL of 647-TmANCs and stirred on a shaker at 1500 rpm for 30 min. The ratio of EDC to FA was optimized based on the loading efficiency of FA on the nanoprobes, in addition to cellular uptake assays. The loading efficiency of FA was determined using the TNBS assay (Thermofisher pierce TNBSA kit), by calculating the number of free amine groups on nanoprobe surface. Using 0.1 M sodium tetraborate buffer, the percentage of FA loading was determined by comparing the number of free amine groups on unfunctionalized versus functionalized probes. The optimal loading was established to be 36%.

All functionalized nanoprobes were characterized similarly to the unfunctionalized ReANCs using DLS to analyze their size distribution and the bicinchoninic acid assay to calculate percentage yield.

### In vitro uptake of functionalized ReANCs

The relative expression levels of CXCR4, CAV1, and FRα were examined in MCF7, 4175TR, and SKOV3 cells using western blots. Target validation was assessed by cellular uptake as measured by flow cytometry. Cells were seeded at 5 × 10^5^ cells per well in a 96-well plate and treated with 10% (v/v) nanoprobes (functionalized or unfunctionalized) for 24 h at 37 °C, 5% CO_2_. Cells were subsequently trypsinized and fixed in 1% paraformaldehyde (PFA) followed by flow cytometry analysis using FACsCalibur™. For fluorescence microscopic imaging, cells were plated at a density of 20,000 cells per well in 8-well borosilicate plates (LabTek) and treated with nanoprobes overnight at 37 °C, 5% CO_2_. Cells were then washed and fixed in 4% PFA and imaged using fluorescence microscopy (Nikon).

### In vivo imaging

Imaging studies were conducted using female homozygous nude mice (Taconic Biosciences, Hudson, NY). For subcutaneous tumor imaging, MCF7 human breast cancer cells, 4175TR human breast cancer cells, or SKOV3 ovarian cancer cells were injected into the dorsal area at 10^7^ cells per site. Animals underwent ReANC administration and whole body SWIR imaging once tumors became palpable. All animal studies were approved by the Institutional Animal Care and use committee (IACUC) of Rutgers University and were performed in accordance with institutional guidelines on animal handling. Animals (*n* = 5/cage) were housed in sterile conditions (sterile disposable cages with sterile bedding, food and water).

### Whole body SWIR imaging

A whole body SWIR imaging system, built in-house as described in previous studies [13, 15, 18], was used for in vivo imaging. Athymic nude mice (Taconic Biosciences, Hudson, NY) were fully anesthetized using 2–3% isoflurane (Butler-Schein, Dublin, OH) and were continuously scanned with a collimated 980 nm laser (output beam collimated to 9.6 mm) to excite nanoprobes. The output optical power within the collimated beam was 1.7 W. Rare-earth emissions were detected with an InGaAs camera (512 × 640 pixels) [28] (640HSX-1.7RT, Sensors Unlimited, Princeton, NJ), equipped with a 25 mm focal length, f/1.4 SWIR lens (SR0907, Stingray Optics, Keene, NH). Three different filter sets were attached to the front threading of the camera lens for discerning distinct ReANC emissions. The filter sets used were: HoANCs: two long-pass 1020 nm (FF01-1020LP, Semrock) and one short-pass 1250 nm filter (89–675, Edmund Optics); ErANCs: two long-pass 1350 nm filters (FELH1350, Thorlabs) and one 1497–1579 nm band-pass filter (FF01–1538/82, Semrock); TmANCs: two long-pass 1350 nm filters (FELH1350, Thorlabs). This system is capable of real-time live animal imaging with a frame exposure time of 33 ms [13]. Images were acquired as .bin video files during scanned illumination and processed using custom Matlab scripts [18].

### In vivo unilateral subcutaneous tumor models

#### Cav-1 positive tumor model

1 × 10^7^ 4175 breast cancer cells (CAV1 receptor positive) were injected into the left dorsal flank of 3–4 week old female, athymic homozygous nude mice (Taconic Biosciences, Hudson, NY). Tumors grew until palpable with tumor volume measured weekly by calipers. Once tumor volume reached around 500 mm^3^, animals received intraperitoneal (i.p.) injections (100 μL, 10 mg/kg dose) of either untargeted (Rh-HoANCs) or targeted (Rh-fHoANCs) holmium nanoprobes, followed by whole body SWIR imaging at 24 h. Animals were sacrificed immediately after imaging and tumors collected for ex vivo analysis.

#### CXCR4 positive tumor model

5 × 10^6^ MCF7 breast cancer cells (CXCR4 positive) were inoculated in the right dorsal flanks of 3–4 week old female, athymic homozygous nude mice (Taconic Biosciences, Hudson, NY) supplemented with 1.5 mg 17β-estradiol pellets (Innovative Research of America) releasing estradiol at a rate of 16.66 μg/day. Tumors grew until palpable with tumor volume measured weekly by calipers. Once tumor volume reached around 500 mm^3^, animals received i.p. injections (100 μl, 10 mg/kg dose) of either untargeted (FITC-ErANCs) or targeted (FITC-fErANCs) erbium nanoprobes followed by whole body SWIR imaging. Animals were sacrificed after imaging and tumors collected for ex vivo analysis.

#### FR-alpha positive tumor model

5 × 10^6^ SKOV3 ovarian cancer cells (Folate Receptor positive) were injected into the left dorsal flanks of 16–17 week old female athymic nude mice (Taconic Biosciences, Hudson, NY). Tumors were allowed to grow until palpable with tumor volume measured weekly by calipers. Once tumor volume reached around 500 mm^3^, animals received i.p. injections (100 μl, 10 mg/kg) of either untargeted (647-TmANCs) or targeted (647-fTmANCs) thulium nanoprobes followed by whole body SWIR imaging. Animals were sacrificed after imaging and tumors collected for ex vivo analysis.

### In vivo bilateral subcutaneous model

1 × 10^7^ 4175TR cells were inoculated into the left dorsal flank and 5 × 10^6^ MCF7 cells were inoculated in the right dorsal flank of 3–4-week-old female athymic nude mice (Taconic Biosciences, Hudson, NY) supplemented with 17β-estradiol pellets releasing estradiol at a rate of 16.66 μg/day. Once tumors reached around 500 mm^3^ in volume, animals were injected with nanoprobes as described above. To assess nanoprobe targeting to 4175 tumors (Fig. 5a), animals were either injected (i.p) with Rh-HoANCs (untargeted) or Rh-fHoANCs (targeted to 4175 tumors) at a dosage of 100 μl, 10 mg/kg. To assess nanoprobe targeting to MCF7 tumors (Fig. 5b), animals were either injected (i.p) with FITC-ErANCs (untargeted) or FITC-fErANCs (targeted to MCF7 tumors), at a dosage of 100 μl, 10 mg/kg. To assess nanoprobe targeting to both MCF7 and 4175 tumors in the same animal (**Supplementary Fig.** **7**), sequential injections (i.p) of Rh-fHoANCs followed by FITC-fErANCs 24 h later were performed.

### SWIR image analysis

Animals were imaged pre- and post- administration of nanoprobes by acquiring a continuous uncompressed video while the illumination beam was scanned over the animal. Custom image processing code (Matlab, Mathworks, Natick, MA) was used to extract the maximum value (12-bit, 0–4095) from each pixel over all frames in the video and save the resulting maximum intensity projection as a .tiff image file. Image processing required manual selection of regions-of-interest (ROIs) around tumors from images of animals acquired under white light. These ROIs were then applied to the corresponding SWIR images and the mean signal intensity for the region was calculated pre- and post- ReANC injection. These values were then used to perform statistical analysis to compare the mean SWIR intensities from each ROI (background-corrected), between experimental groups receiving untargeted and targeted nanoprobes.

### Ex vivo tumor imaging

All tumor-bearing animals were sacrificed at experimental end points according to the Institutional Animal Care and use committee (IACUC) of Rutgers University guidelines, 24-h post nanoprobe injection and tumors were excised. Briefly, animals were euthanized by compressed carbon-di-oxide (CO_2_) exposure using systems displace 10–30% of chamber volume per minute followed by cervical dislocation. Ex vivo SWIR imaging was performed on tumors along with wide-field fluorescence imaging (MS FX PRO, Carestream Molecular Imaging). Tumor samples were then flash frozen for microscopic analysis.

### Ex vivo confocal imaging

Flash frozen tumor samples were cryosectioned at 50 μm onto microscope slides and then imaged on a Zeiss LSM 780 confocal microscope, equipped with a spectral detector (Quasar) for taking lambda stacks. Rhodamine-B, FITC, and Alexa Fluor 647 dissolved in ethanol were used to build reference spectra with the spectral detector, with an untreated tumor used a control for tissue autofluorescence. An 18-channel lambda-stack was acquired for each reference sample, covering an emission range of 498–695 nm, using 488 nm, 561 nm and 647 nm lasers for excitation. Once reference spectra were obtained, online fingerprinting was used for spectral unmixing of all four signals (Rhodamine-B, FITC, Alexa Fluor 647, and tissue autofluorescence). Samples were imaged using a 10x objective, and tile-scanning was implemented to scan each tissue section.

### Statistical analysis

For all in vivo studies, mice were randomly assigned to each experimental group with investigators unblinded to the acquisition and analysis of data. Grubb’s test for outliers was used to determine inclusion or exclusion of data within groups for all data sets. Statistical tests were selected based on the normality of the distribution of the mean SWIR intensity values, sample size, and the similarity in variance between groups. Statistical significance of the normal populations was determined using the Mann-Whitney U test and Welch’s t-test.

## Results

### Synthesis and characterization of multi-colored nanoprobes

RE nanoprobes synthesized with Ho, Er, and Tm as core dopants produced distinct emission signatures in the SWIR spectral region (**Supplementary Fig.** **1**). Following encapsulation in human serum albumin, HoANCs, ErANCs, and TmANCs were generated with diameters ranging from 133 to 161 nm (**Supplementary Fig.** **2**). The visible light emitting fluorophores rhodamine-B (Rh), fluorescein isothiocyanate (FITC), and Alexa Fluor 647 (647) were incorporated within HoANCs, ErANCs, and TmANCs, respectively, generating Rh-HoANCs, FITC-ErANCs, and 647-TmANCs. The visible fluorescence emission relative to background produced by the albumin nanoprobes was greatest for Rh-HoANCs, followed by FITC-ErANCs, and finally 647-TmANCs (**Supplementary Fig.** **3**). Cancer targeted nanoprobes were either adsorbed (AMD3100 targeting CXCR4 and Diadzein targeting Caveolin-1) or chemically crosslinked (folic acid targeting folate receptor) to the surface of the albumin nanocomposites. Physical adsorption to the surface was validated through SEM in our previous studies [15] and chemical crosslinking was validated determining available free amine groups as shown in previous studies [28].

### In vitro target validation of functionalized multi-color probes

**Fig. 2:**
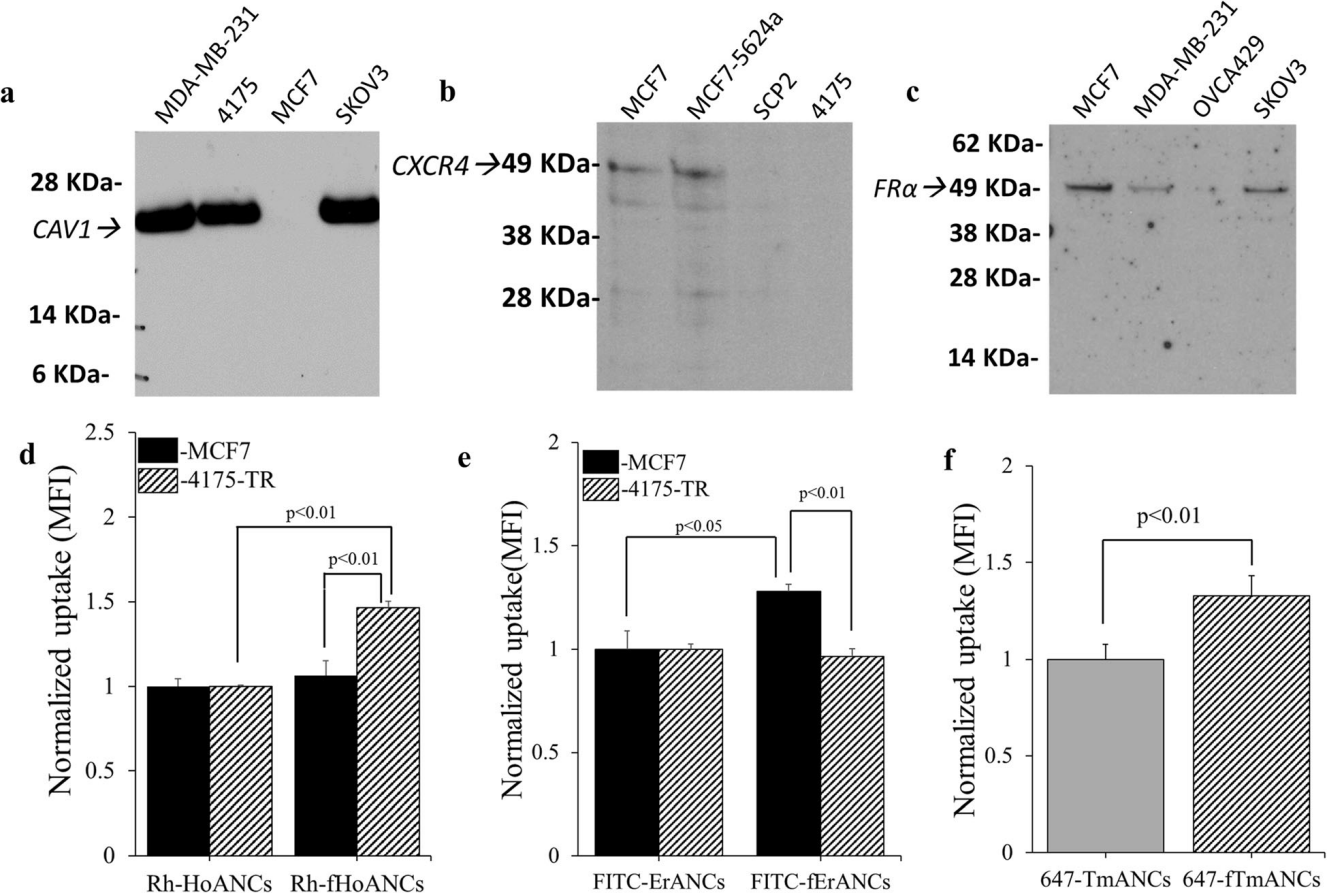
Targeting Validation of functionalized multi-color nanoprobes in vitro. Western blots indicate the relative expression levels of three different cancer biomarkers: **a** The 4175TR cell line shows increased CAV1 receptor expression relative to the MCF7 cell line. **b** MCF7 cells show higher CXCR4 expression than 4175TR cells. **c** SKOV3 ovarian cancer cell line used in this study were determined to be FR positive by immunoblotting. The western blots in **a**, **b** an **c** panels have been cropped to highlight the protein of interest in each panel. Panel **a**, **b** and **c** show western blots of caveolin-1, CXCR4 and folate receptor- alpha respectively. The full-length western blots are presented in **Supplementary Fig.** 9. **d** Flow cytometry shows a statistically significant increase in uptake of CAV1-targeted nanoprobes (Rh-fHoANCs) compared to untargeted nanoprobes (Rh-HoANCs) in CAV1-expressing 4175TR cells. No significant increase was observed in uptake of CAV1-targeted versus untargeted nanoprobes in CAV1-negative MCF7 cells. **e** CXCR4-targeted nanoprobes (FITC-fErANCs) showed significantly increased uptake in CXCR4-expressing MCF7 cells compared to untargeted nanoprobes (FITC-ErANCs). No significant increase was observed in uptake of CXCR4-targeted versus untargeted nanoprobes in CXCR4-negative 4175TR cells. **f** A statistically significant increase in uptake of FRα-targeted nanoprobes (647-fTmANCs) compared to untargeted nanoprobes (647-TmANCs) was observed in FRα-expressing SKOV3 cells. For panel **d**, **e** and **f** student t-test was used to determine statistical significance between two groups

As shown in Fig. 2a, we found essentially exclusive expression of CAV1 on 4175TR cells compared to MCF7 cells. MCF7 cells exhibited much higher expression of CXCR4 compared to 4175TR cells (Fig. 2b) and SKOV3 cells showed expression of FRα (Fig. 2c). The ability of our functionalized nanoprobes to specifically target these differentially regulated receptors was evaluated by flow cytometry. There was a statistically significant increase in uptake of CAV1-targeted nanoprobes (Rh-fHoANCs) compared to untargeted nanoprobes (Rh-HoANCs) in CAV1-expressing 4175TR cells (Fig. 2d). The western blots represented in Fig. 2 (a), (b) and (c) presenting proteins caveolin-1, CXCR4 and folate receptor alpha respectively, have been cropped to focus on the protein of interest. The corresponding full-length western blots are shown in **Supplementary Fig.** **9**. The cropped portion represented in Fig. 2 (a), (b) and (c) are highlighted by red arrows in **Supplementary Fig.** **9****.** No significant increase was observed in uptake of CAV1-targeted versus untargeted nanoprobes in CAV1-negative MCF7 cells (Fig. 2d). Similarly, we observed a statistically significant increase in uptake of CXCR4-targeted nanoprobes (FITC-fErANCs) compared to untargeted nanoprobes (FITC-ErANCs) in CXCR4-expressing MCF7 cells (Fig. 2e). No significant increase was observed in uptake of CXCR4-targeted versus untargeted nanoprobes in CXCR4-negative 4175TR cells. We also saw a statistically significant increase in uptake of FRα-targeted nanoprobes (647-fTmANCs) compared to untargeted nanoprobes (647-TmANCs) in FRα-expressing SKOV3 cells (Fig. 2f). Optimal targeting ligand concentrations of 3.93 × 10^− 4^ M daidzein and 1.25 × 10^− 6^ M AMD3100 were estimated for Rh-fHoANCs and FITC-fErANCs respectively, by measuring cellular uptake of targeted vs untargeted nanoprobes over a range of ligand concentrations (**Supplementary Fig.** **4**). Uptake of targeted and untargeted nanoprobes to MCF7 and 4175TR cells was examined using confocal microscopy, showing increased uptake of Rh-fHoANCs and FITC-fErANCs nanoprobes by their targeted cell lines (**Supplementary Fig.** **5**).

### In vivo targeting of nanoprobes in subcutaneous tumor bearing mice

**Fig. 3:**
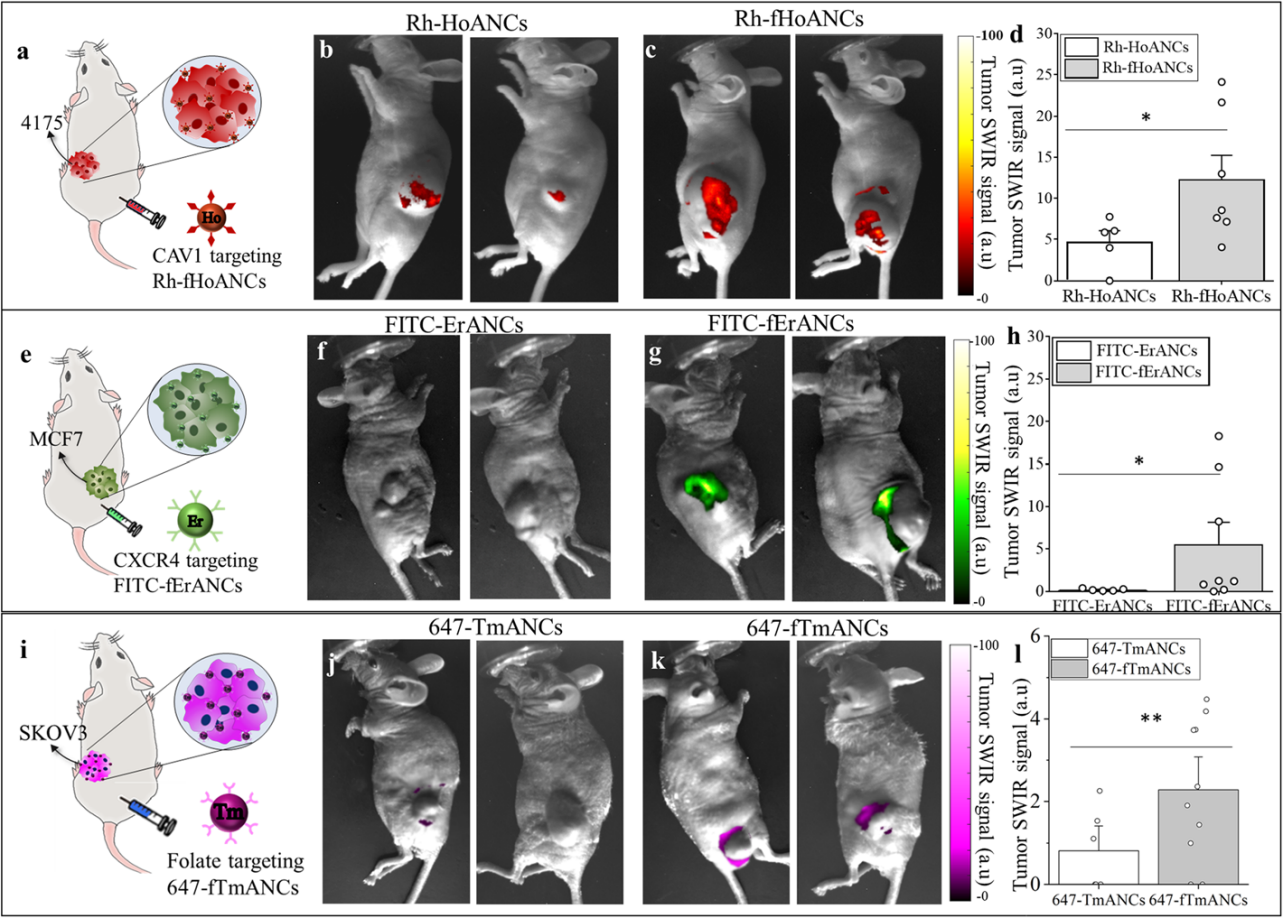
Multi-Color Nanoprobe Targeting Validation in vivo. **a** Mice bearing subcutaneous 4175TR tumors received CAV1 targeted (Rh-fHoANC) and untargeted (Rh-HoANC) holmium-doped nanoprobes. Representative images of **b** Rh-HoANC and **c** Rh-fHoANC accumulation at 6-weeks post-inoculation. **d** SWIR signal intensities show brighter emissions from animals injected with targeted (*n* = 7) compared to untargeted (*n* = 5) nanoprobes. **e** Mice bearing subcutaneous MCF7 tumors received CXCR4 targeted (FITC-fErANC) and untargeted (FITC-ErANC) erbium-doped nanoprobes. Representative images of **f** FITC-ErANC and **g** FITC-fErANC accumulation. **h** SWIR signal intensities show brighter emissions from animals injected with targeted (*n* = 8) compared to untargeted (*n* = 5) nanoprobes. **i** Mice bearing subcutaneous SKOV3 tumors received folate receptor targeted (647-fTmANC) and untargeted (647-TmANC) thulium-doped nanoprobes. Representative images of **j** 647-TmANC and (k) 647-fTmANC accumulation. **l** SWIR signal intensities show brighter emissions from animals injected with targeted (*n* = 10) compared to untargeted (*n* = 6) nanoprobes. Bar graphs in panels **d**, **h**, and **l** represent the mean ± s.e.m. for each group. *p < 0.07, determined by a two-tailed Mann Whitney U-test; **p < 0.05 determined by Welch’s two-tailed t-test. Panels **b**, **c**, **f**, **g**, **j**, and **k** each present two representative animals from each experimental group

The ability of Rh-fHoANCs to target a specific tumor biomarker was evaluated in mice bearing subcutaneous 4175TR tumors (overexpressing CAV1 receptor) (Fig. 3a). Whole body SWIR imaging was performed 24-h post injection with either targeted or untargeted nanoprobes (Fig. 3b, c). Quantitative image analysis showed a statistically significant increase (~ 2-fold) in SWIR intensity at the tumor site in animals receiving targeted nanoprobes compared to those receiving untargeted nanoprobes (Fig. 3d). In parallel, the targeting ability of FITC-fErANCs was validated in mice bearing subcutaneous MCF7 tumors (overexpressing CXCR4 receptor) (Fig. 3e). Whole body SWIR imaging was performed 24-h post injection with either targeted or untargeted nanoprobes (Fig. 3f, g). Quantitative image analysis showed a statistically significant increase (~ 5-fold) in SWIR intensity at the tumor site in animals receiving targeted nanoprobes compared to those receiving untargeted nanoprobes (Fig. 3h). Similarly, the targeting ability of 647-fTmANCs was validated in mice bearing subcutaneous SKOV3 tumors (overexpressing Folate Receptor alpha) (Fig. 3i). Whole body SWIR imaging was performed 24-h post injection with either targeted or untargeted nanoprobes (Fig. 3j, k). Quantitative image analysis showed a statistically significant increase (~ 2.75-fold) in SWIR intensity at the tumor site in animals receiving targeted nanoprobes compared to those receiving untargeted nanoprobes (Fig. 3l).

### Ex vivo macro- and microscopic imaging of tumors

**Fig. 4:**
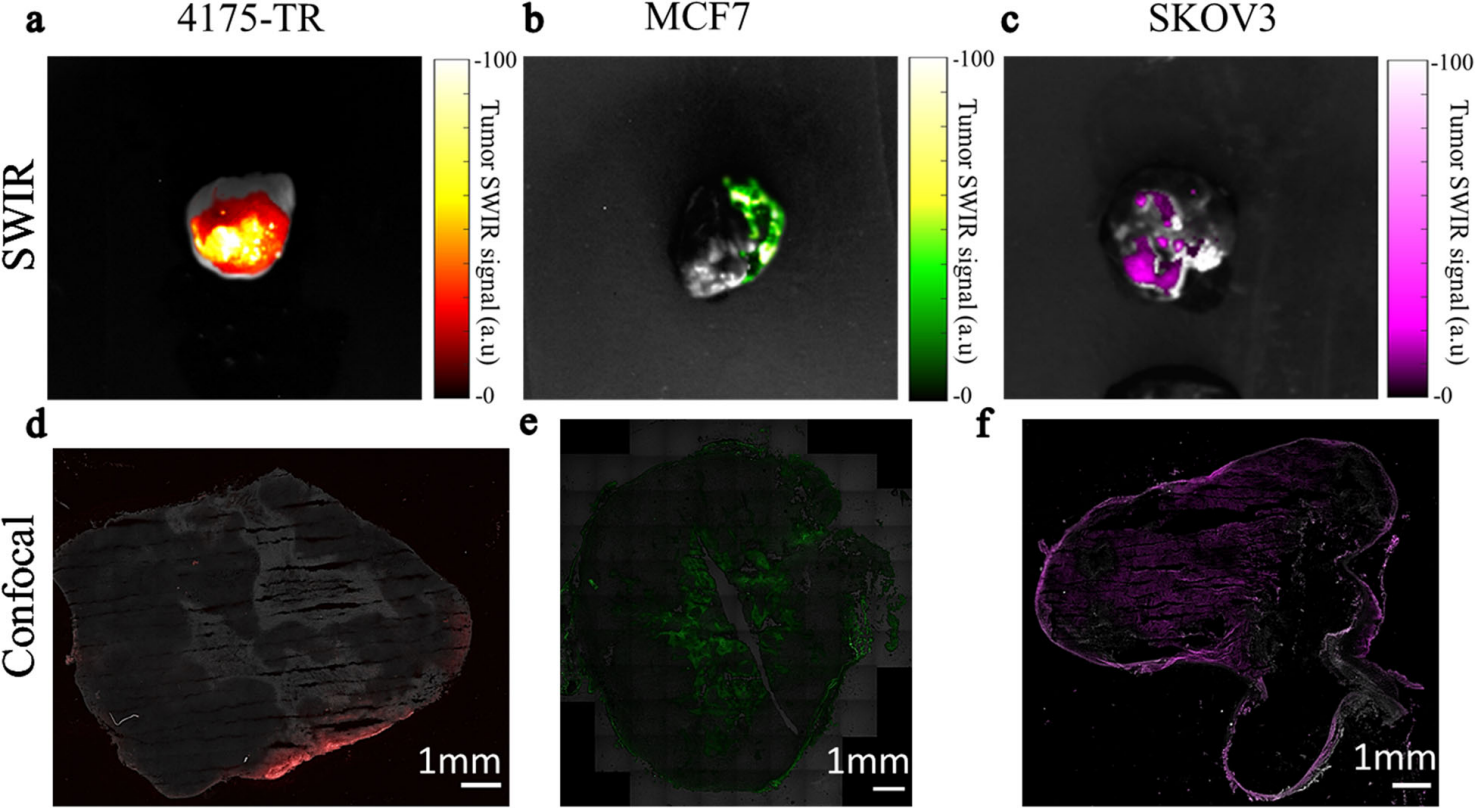
Ex vivo analysis of multi-color probe uptake. Tumors from animals injected with targeted nanoprobes were excised at 24 h post-injection and imaged on macro- and microscopic imaging platforms. **a** SWIR and **d** confocal imaging of Rh-fHoANCs in a 4175TR tumor, **b, e** FITC-fErANCs in a MCF7 tumor, and **c, f** 647-fTmANCs in a SKOV3 tumor. Confocal images **d, e, f** were obtained by measuring the signal from the visible fluorophores loaded into each probe (rhodamine, FITC, Alexa Fluor 647, respectively) Panels **b**, **c**, **f**, **g**, **j**, and **k** each present two representative animals from each experimental group.

Ex vivo SWIR imaging (Fig. 4a) and visible fluorescence imaging (**Supplementary Fig.** **6****a**) of resected 4175 tumors detected the presence of holmium and rhodamine-B respectively. Tumor sections imaged with confocal microscopy also showed accumulation of Rh-fHoANCs as shown by increased rhodamine intensity at the tumor periphery (Fig. 4d). Ex vivo SWIR imaging **(**Fig. 4b**)** and visible fluorescence imaging (**Supplementary Fig.** **6****b**) of resected MCF7 tumors detected the presence of erbium and FITC respectively. Tumor sections imaged with confocal microscopy also showed accumulation of FITC-fErANCs as shown by increased FITC intensity at the tumor periphery and within the core (Fig. 4e). Ex vivo SWIR imaging **(**Fig. 4c**)** and visible fluorescence imaging (**Supplementary Fig.** **6****c**) of resected SKOV3 tumors detected the presence of thulium and Alexa Fluor 647 respectively. Tumor sections imaged with confocal microscopy also showed accumulation of 647-fTmANCs as shown by increased Alexa Fluor 647 intensity at the tumor periphery and within the mass (Fig. 4f).

### In vivo targeting of nanoprobes in a bilateral subcutaneous tumor model

**Fig. 5:**
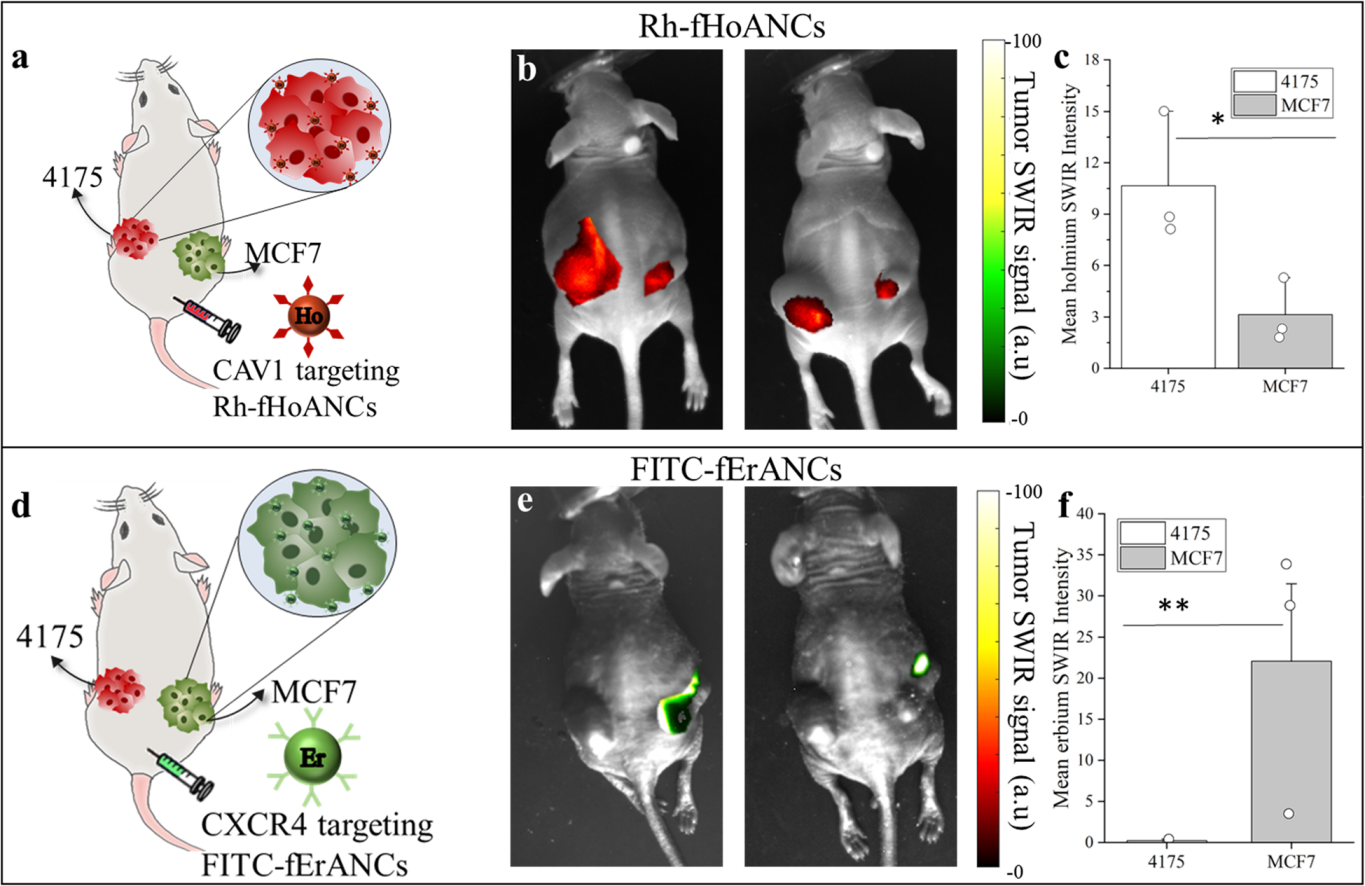
Biomarker Specific Accumulation of Nanoprobes. **a** Mice bearing molecularly-distinct bilateral subcutaneous tumors were injected with CAV1-targeted Rh-fHoANCs nanoprobes. **b** Images of two representative animals show higher accumulation in the CAV1-expressing 4175 tumor (left dorsal flank) compared to the MCF7 tumor (right dorsal flank). **c** The mean SWIR emission intensity from 4175 tumors was significantly higher than from the MCF7 tumors. **d** Mice bearing bilateral tumors were injected with MCF7 targeted FITC-fErANCs nanoprobes. **e** Images of two representative animals show higher accumulation in the CXCR4-expressing MCF7 tumor (right dorsal flank) compared to the 4175 tumor (left dorsal flank) tumors. **f** The mean SWIR emission intensity from MCF7 tumors was significantly higher than from 4175 tumors. Bar graphs in panels **c** and **f** represent the mean ± s.e.m. for each group (*n* = 3 in each). *p < 0.06 determined by Welch’s two-tailed t- test; **p = 0.1 determined by the Mann-Whitney U-test

We investigated the targeting specificity of functionalized nanoprobes in mice bearing bilateral tumors that differ in their biomarker expression levels. These tumors were formed from MCF7 cells (CXCR4 positive) in the right dorsal flank and 4175TR cells (CAV1 positive) in the left dorsal flank. Animals injected with CAV1-targeted Rh-fHoANCs (Fig. 5a) showed approximately 3-fold brighter SWIR signal in 4175TR tumors compared to MCF7 tumors (Fig. 5b, c). Animals injected with CXCR4-targeted FITC-fErANCs (Fig. 5d) showed almost 20-fold increase in SWIR signal from the MCF7 tumors compared to 4175 tumors (Fig. 5e, f).

Finally, in a preliminary study, we demonstrated the ability to perform simultaneous detection of two different biomarkers in the same animal using multi-color imaging. Animals bearing 4175 tumors in the left flank and MCF7 tumors in the right flank were injected with spectrally distinct nanoprobes targeted to biomarkers specific for each tumor. CAV1-targeted Rh-fHoANCs were administered first, with CXCR4-targeted FITC-fErANCs administered 24-h later. SWIR imaging at 24-h and 36-h time points indicated differential localization of nanoprobes based on their distinct SWIR emissions, with increased accumulation of CAV1 targeted nanoprobes in the 4175TR tumor (**Supplementary Fig.** **7****b**), and increased accumulation of CXCR4 targeted nanoprobes in the MCF7 tumor (**Supplementary Fig.** **7****d**).

## Discussion

Chemoresistance [29], failure of targeted therapy [30], and an inability to predict immunotherapy responses [31] are major challenges in clinical oncology. Each of these areas would benefit from a platform that can longitudinally interrogate multiple biomarkers that are informative of disease state in small animal models. The ability to label and non-invasively study multiple tumor subtypes and interacting elements of the microenvironment and immune system would lead to a richer understanding of topics including tumor heterogeneity and immune cell-tumor interactions. The goal of this study was to advance our previous work on single color, SWIR-based surveillance nanotechnology towards a proof-of-concept, multi-color, in vivo imaging platform by developing a library of biomarker-specific nanoprobes that can discern distinct cellular subsets.

We synthesized three biomarker-specific nanoprobes, each with distinct SWIR emission spectra when illuminated with NIR light (Figs. 3, 4, 5). The use of albumin as an encapsulating agent confers biocompatibility and allows a range of targeting ligands to be added either by physical adsorption or by chemical conjugation. The ability of albumin for physically binding by adsorption and presenting targeting ligands is somewhat remarkable and may reflect the natural role for this protein in binding and sequestering drugs and other organic compounds. Incorporation of conventional fluorophores that emit in the visible and far-red spectral regions allows for ex vivo validation of in vivo observations on standard commercial microscopy platforms (Fig. 4d-f**, Supplementary Fig.** **4**). We established the optimal loading conditions for three selected targeting ligands by FACS-based target validation (Fig. 2). Additionally, targeting specificity of these probes was compared through competitive inhibition assays using excess of the SDF-1 ligand, a caveolin-1 specific antibody and excess folic acid for inhibition of binding of AMD 3100 functionalized ReANCs to CXCR-4, for daidzein functionalized ReANCs to Caveolin-1 and folic acid functionalized ReANCs to folate receptor respectively.

In this study, probes were developed to target three very different cancer biomarkers: CXCR4, which is overexpressed in a wide variety of cancers and is associated with an aggressive, metastatic phenotype; CAV1, whose role in oncogenesis is heavily context-dependent; and the folate receptor, which is noted for frequent overexpression in ovarian and breast cancers. The ability of these targeted nanoprobes to discern distinct tumor populations was demonstrated in subcutaneous tumor models. Each of the targeted nanoprobes exhibits higher accumulation in the tumors overexpressing the corresponding oncogenic biomarker, compared to their untargeted counterparts. In a bilateral tumor model, we show high specificity in targeting AMD3100 functionalized probes to MCF7 tumors and daidzein-targeted probes to 4175 tumors (Fig. 5). In a preliminary bilateral tumor model, animals sequentially injected with biomarker-specific nanoprobes demonstrated preferential accumulation of target specific probes to their respective tumors: CXCR4 targeted probes to MCF7 cells and CAV1 targeted probes to 4175TR cells. Taken together, these studies demonstrate the applicability of the targeted SWIR imaging approach across a variety of tumors and biomarkers.

The novel design that combines the SWIR emissions for macroscopic imaging with the traditional fluorophore emissions for microscopic imaging allows for ex vivo validation and opens the possibility for future molecular signature analysis of multiple cellular subpopulations at a microscopic level. Additionally, we have shown ex-vivo microscopic validation of nanoprobe accumulation by confocal microscopy of tumor sections, which provides a foundation for conventional histopathological evaluations in clinical practice when the technology is translated for human use.

The study highlights the capability of the SWIR emitting nanoprobes to discern biomarker-specific single tumors. However, the translational value lies in the ability to potentially distinguish biomarker-specific cellular subsets within a single tumor to reveal the molecular nature of intra-tumor heterogeneity. The lack of a mouse model that phenocopies human intra-tumor heterogeneity has prevented us from presenting this valuable information. Future studies will focus on engineering of such a tumor and developing a mouse model that will bring the application of the biomarker-specific nanoprobes closer to translatability.

## Conclusions

Several studies have explored multi-color real time imaging for tumor heterogeneity mapping. These studies highlight the promise for the use of imaging biomarkers such as those used in this study for diagnosis, to be incorporated in drug development process to unravel changes in molecular pathways in response to drugs [5]. The power of imaging biomarkers integrated with a contrast agent that has deeper penetration potential than the fluorophores shown thus far in established studies [32–44] will be beneficial for numerous applications in cancer research.

Tumor heterogeneity can be attributed to: a) spatial heterogeneity; b) temporal heterogeneity as a result of either natural progression of disease or treatment; c) population-based heterogeneity and/or d) heterogeneity based on micro-environmental changes [45–48]. Precision imaging of cancer heterogeneity will play an important role in determining optimal therapeutic strategies. This study has demonstrated the ability of our multi-colored engineered probes to interrogate different tumor subsets by targeting specific biomarkers in vivo*,* which will pave way for a non-invasive optical signature mapping system for tumors. Future studies will focus on refining the ReANC chemistry to yield brighter nanoprobes with emissions at additional spectral locations within the SWIR region [49]. This will potentially enable dense multiplexed imaging of tumor biomarkers, extracellular matrix components, and immune cells to study important topics in preclinical models.

## Supplementary Information

**Additional file 1 Figure S1.** SWIR emissions of rare earths with varying dopant chemistries. **Figure S2.** Size characterization of untargeted and targeted multi-colored nanoprobes. **Figure S3.** Increased fluorescence in nanoprobes loaded with fluorescent dyes in their respective excitation regions. **Figure S4.** Cellular uptake of targeted vs untargeted nanoprobes with varying ligand loading concentrations. **Figure S5.** Confocal imaging of cells with targeted vs untargeted nanoprobes. **Figure S6.** Ex vivo imaging of tumors. **Figure S7.** Biomarker specific accumulation of targeted nanoprobes in a single animal following sequential injection. **Figure S8.** Animal weights monitored through study course. **Figure S9.** Full length western blots for Caveolin-1, CXCR4 and Folate receptor alpha proteins.

## Data Availability

The data sets generated during and/or analyzed in this study are available from the corresponding author on reasonable request.

## Abbreviations

SWIR: Short-wave infrared
NIR: Near-infrared
FITC: Fluorescein isothiocyanate
Rh: Rhodamine
RE: Rare-earth
Ho-RE: Holmium-doped rare-earth
Er-RE: Erbium-doped rare-earth
Tm-RE: Thulium-doped rare-earth
ReANCs: Rare-earth-doped albumin nanocomposites
HoANCs: Holmium-doped rare-earth albumin nanocomposites
ErANCs: Erbium-doped rare-earth albumin nanocomposites
TmANCs: Thulium-doped rare-earth albumin nanocomposites
fReANCs: Functionalized rare-earth-doped albumin nanocomposites
FITC-fErANCs: Fluorescein-labelled, erbium-doped, AMD3100 functionalized rare-earth albumin nanocomposites
Rh-fHoANCs: Rhodamine-labelled, holmium-doped, daidzein functionalized rare-earth albumin nanocomposites
647-fTmANCs: Alexa Fluor 647-labelled, thulium-doped, folic acid functionalized rare-earth albumin nanocomposites
CXCR-4: C-X-Chemokine Receptor-4
CAV1: Caveolin − 1 receptor
FRα: Folate receptor alpha
DLS: Dynamic Light Scattering
PDI: Polydispersity index
BCA: Bicinchoninic acid assay
PFA: Paraformaldehyde
EDC: 1-ethyl-3-(3-dimethylaminopropyl)carbodiimide
