# Supplementary material for: Shortwave infrared emitting multicolored nanoprobes for biomarker-specific cancer imaging in vivo
Source: BMC Cancer. 2020 Nov 10;20:1082. doi: 10.1186/s12885-020-07604-8 (PMC7654009; doi:10.1186/s12885-020-07604-8)
Supplement: Supplementary file 1 — Additional file 1 Figure S1. SWIR emissions of rare earths with varying dopant chemistries. Figure S2. Size characterization of untargeted and targeted multi-colored nanoprobes. Figure S3. Increased fluorescence in nanoprobes loaded with fluorescent dyes in their respective excitation regions. Figure S4. Cellular uptake of targeted vs untargeted nanoprobes with varying ligand loading concentrations. Figure S5. Confocal imaging of cells with targeted vs untargeted nanoprobes. Figure S6. Ex vivo imaging of tumors. Figure S7. Biomarker specific accumulation of targeted nanoprobes in a single animal following sequential injection. Figure S8. Animal weights monitored through study course. Figure S9. Full length western blots for Caveolin-1, CXCR4 and Folate receptor alpha proteins. [file 12885_2020_7604_MOESM1_ESM.docx]

**Supplementary Figure 1:**

**
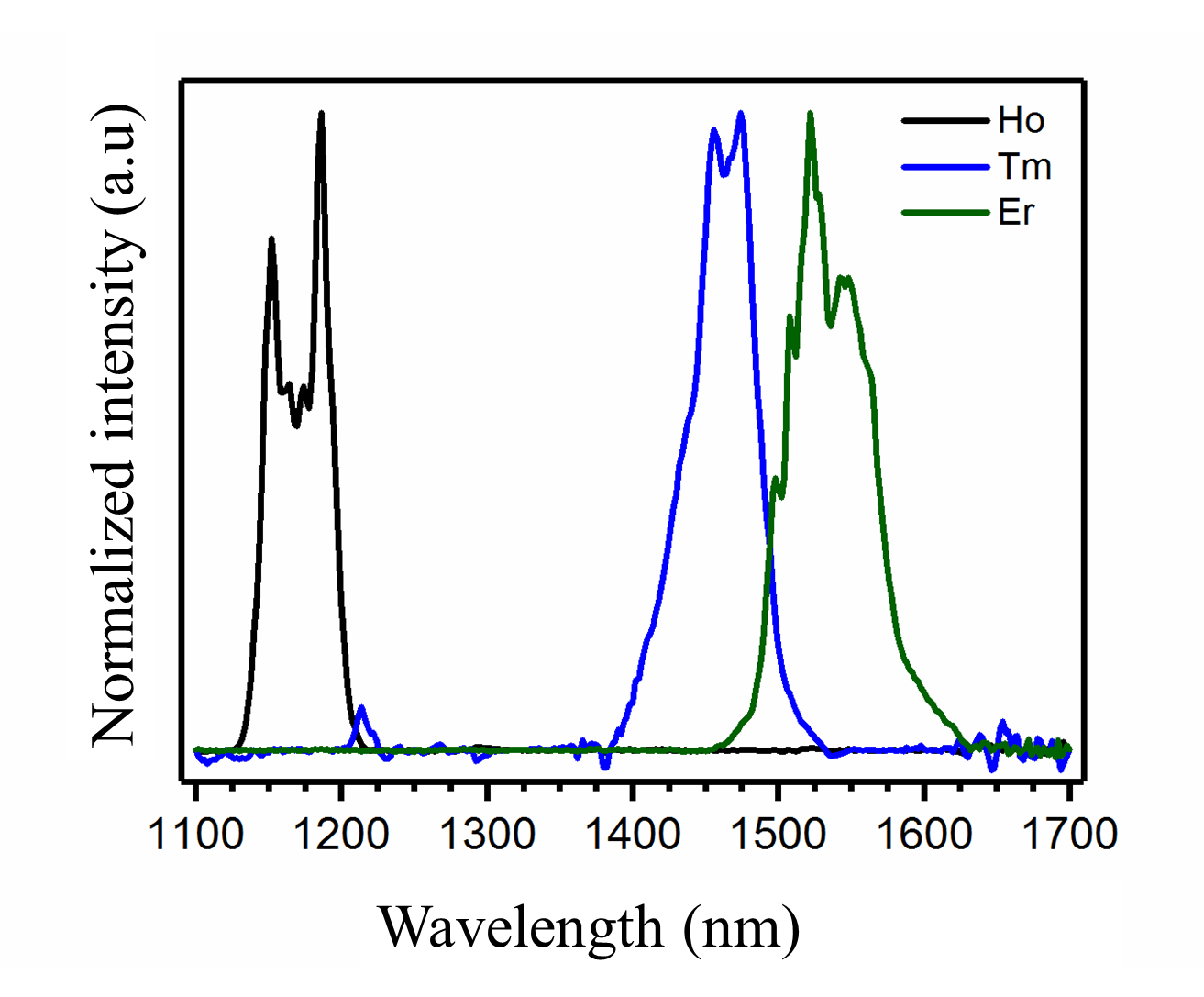
**

**Supplementary Figure 1: SWIR emissions of rare earths with varying dopant chemistries:** When excited at 980 nm, Holmium (Ho), thulium (Tm), and erbium (Er) doped rare-earth nanoprobes emit distinct spectra in the SWIR region, with peaks at 1185 nm, 1475 nm, and 1525 nm respectively.

**Supplementary Figure 2:**

**
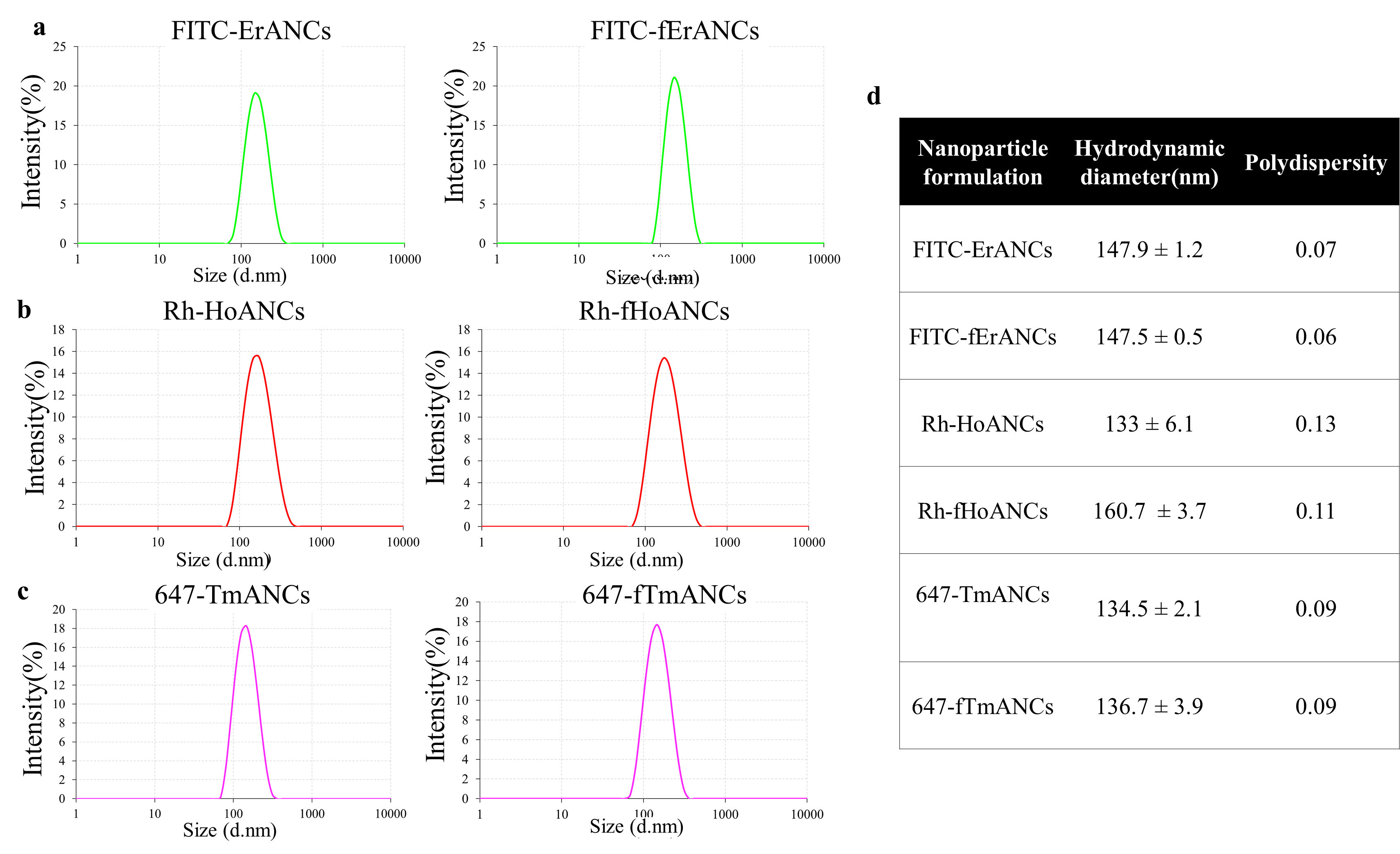
**

**Supplementary Figure 2: Size characterization of untargeted and targeted multi-colored nanoprobes.**  The hydrodynamic diameters of different nanoprobe formulations were characterized using dynamic light scattering (DLS). (a) The hydrodynamic diameters of FITC-loaded untargeted (FITC-ErANC) and targeted (FITC-fErANC) nanoprobes were 147.9 nm and 147.5 nm, respectively. (b) The hydrodynamic diameters of rhodamine-loaded untargeted (Rh-HoANCs) and targeted (Rh-fHoANCs) nanoprobes were measured to be 133.0 nm and 160.7 nm, respectively. (c) The hydrodynamic diameters of Alexa Fluor 647-loaded untargeted (647-TmANCs) and targeted (647-fTmANCs) nanoprobes were measured to be 134.5 nm and 136.7 nm, respectively.

**Supplementary Figure 3:**

**
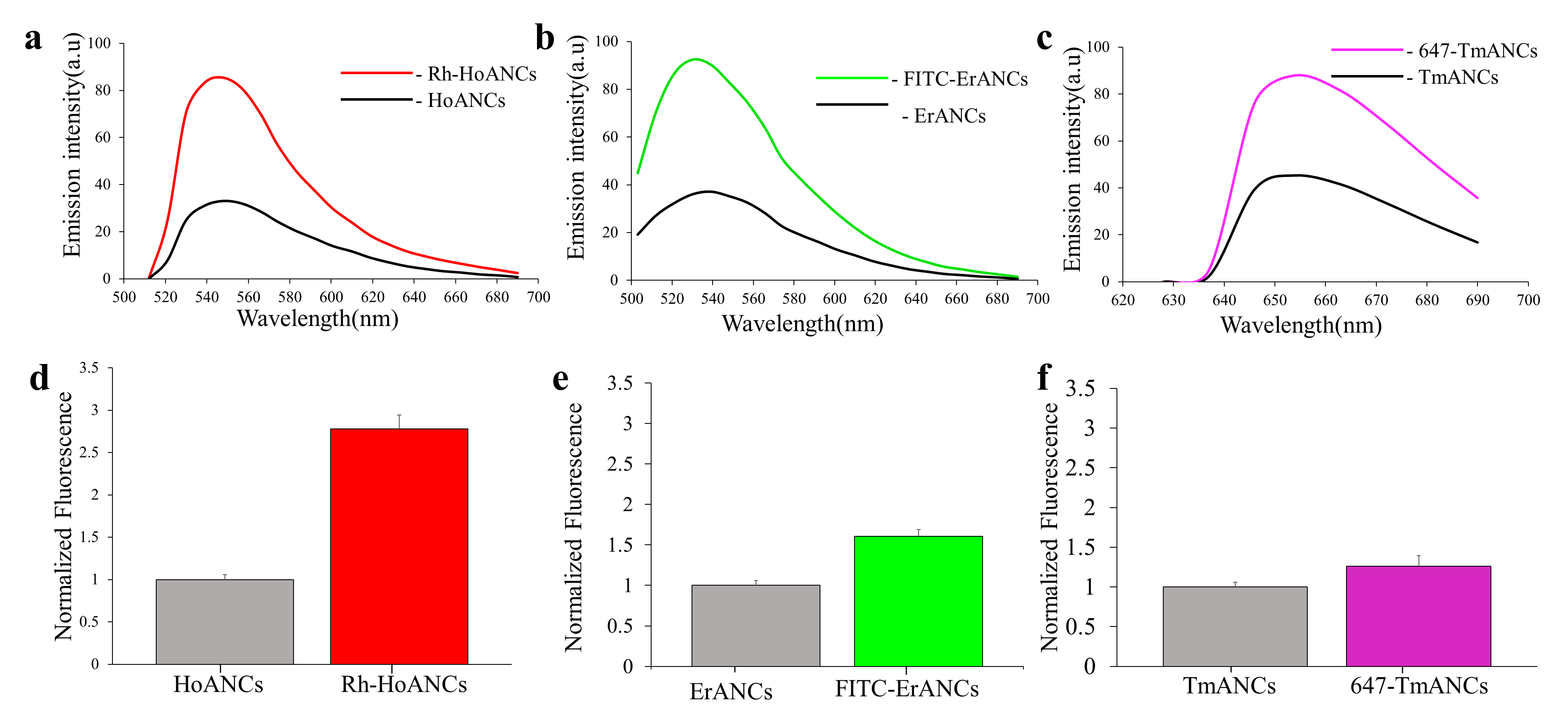
**

**Supplementary Figure 3: Increased fluorescence in nanoprobes loaded with fluorescent dyes in their respective excitation regions:** Nanoprobes(a) Rh-HoANCs, (b) FITC-ErANCs, and (c) 647-TmANCs show higher fluorescence intensity compared to unmodified ReANCs in FITC, rhodamine and alexa-647 channels respectively. Quantification of emissions from fluorophore-loaded ReANCs using a plate reader showed increased intensities compared to unmodified ReANCs in each case (d-f).

**Supplementary Figure 4:**


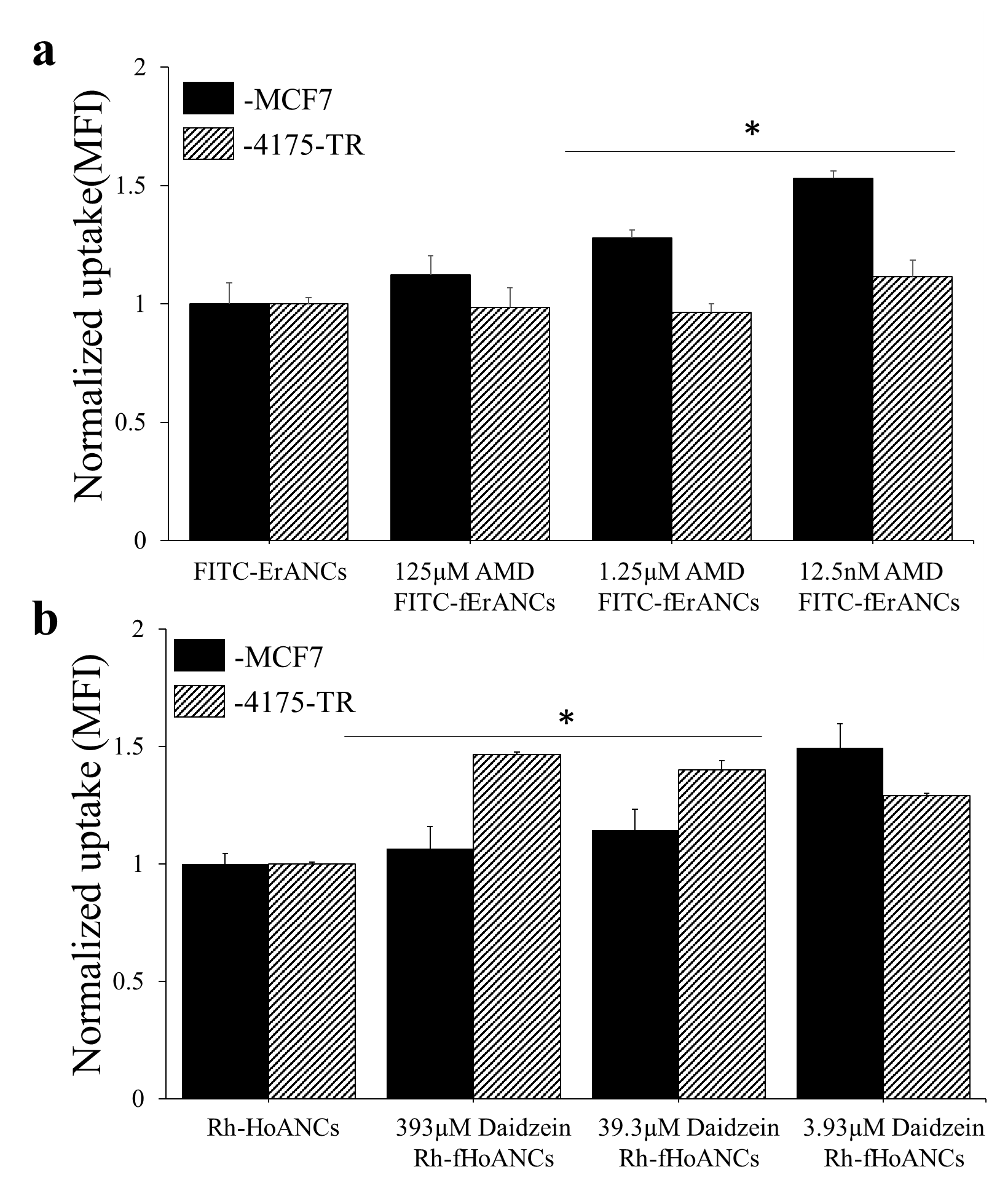


**Supplementary Figure 4: Cellular uptake of targeted vs untargeted nanoprobes with varying ligand loading concentrations:** Flow cytometry was used to determine the optimal concentration of targeting ligands for each ReANC formulation. (a) MCF7 and 4175 cells were treated with FITC-fErANCs loaded with varying concentrations of AMD3100. Mean fluorescence intensity normalized to cells treated with untargeted particles showed an increase in uptake of CXCR4-targeted nanoprobes (FITC-fErANCs) compared to untargeted nanoprobes (FITC-ErANCs) in CXCR4-expressing MCF7 cells.  No significant increase was observed in uptake of CXCR4-targeted versus untargeted nanoprobes in CXCR4-negative 4175 cells. (b) Similarly, when MCF7 and 4175 cells treated with Rh-fHoANCs loaded with varying concentrations of Daidzein. Targeted nanoprobes (Rh-fHoANCs) showed increased uptake in CAV1-positive 4175 cells compared to untargeted nanoprobes (Rh-HoANCs). In contrast, CAV1-negative MCF7 cells did not show any significant increase in uptake between targeted and untargeted probes. *p < 0.05 (Student’s t-test).

**Supplementary Figure 5:**

**
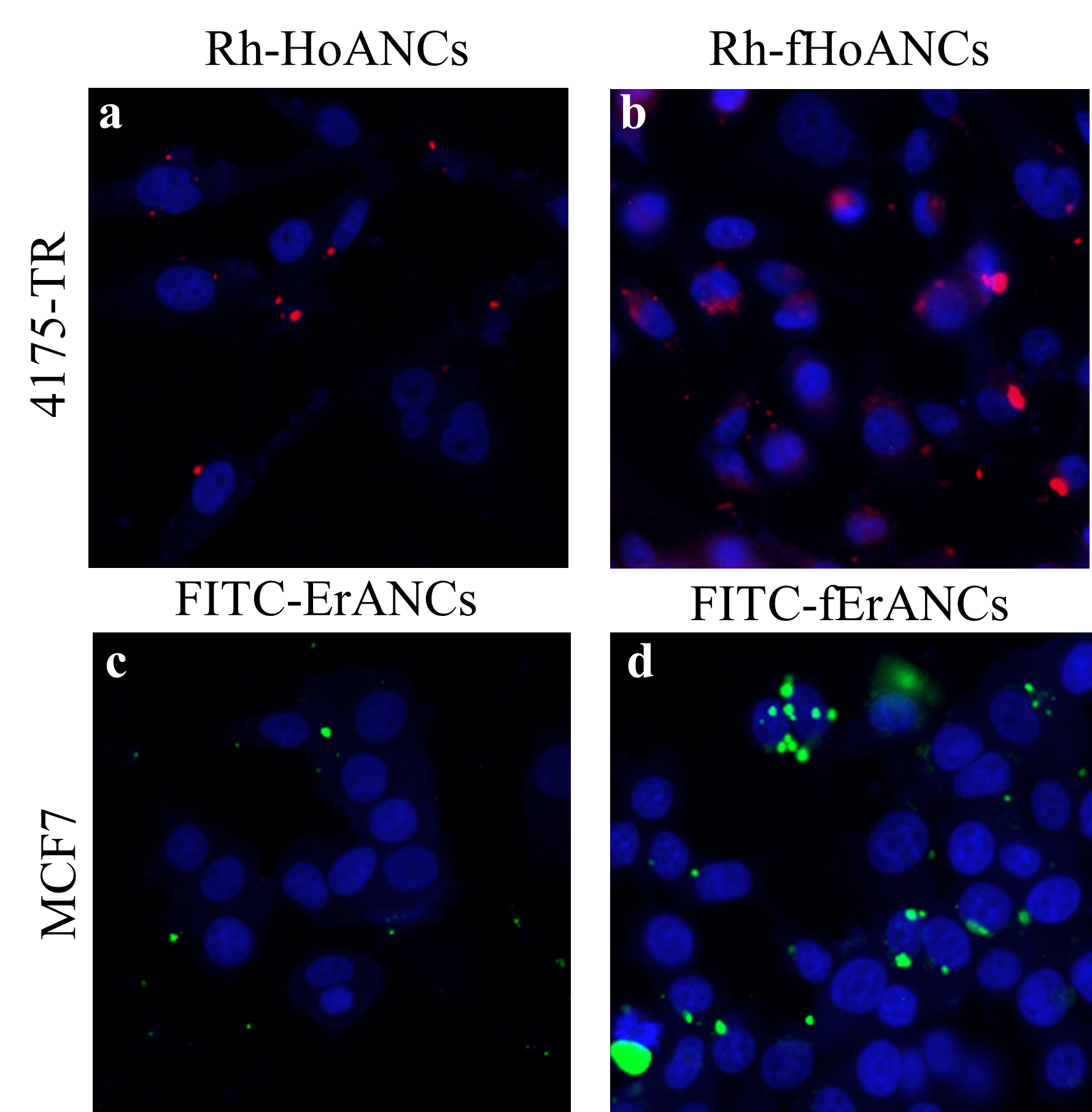
**

**Supplementary Figure 5: Confocal imaging of cells with targeted vs untargeted nanoprobes:** (a,b) 4175TR (CAV1+) cells were treated with untargeted (Rh-HoANCs) and targeted (Rh-fHoANCs) nanoprobes. Confocal fluorescence intensities in the rhodamine channel showed increased nanoprobe uptake with targeted (b) compared to untargeted nanoprobes (a). MCF7 (CXCR4+) cells were treated with untargeted (FITC-ErANCs) and targeted (FITC-fErANCs) nanoprobes. Confocal fluorescence intensities in the FITC channel showed increased nanoprobe uptake with targeted (d) compared to untargeted nanoprobes (c).

**Supplementary Figure 6:**

**
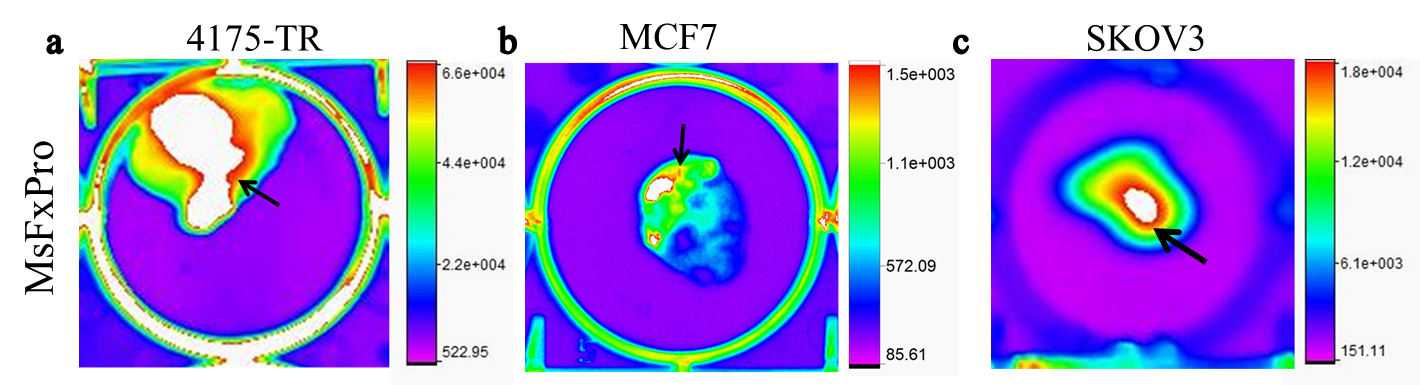
**

**Supplementary Figure 6: Ex vivo imaging of tumors:** Animals injected with targeted nanoprobes were sacrificed 24 hours post ReANC injection. Fluorescent emissions from resected tumors were imaged to show presence of Rh-fHoANCs in 4175 (a), FITC-fErANCs in MCF7 (b) and 647-fTmANCs in SKOV3 tumors (c).

**Supplementary Figure 7:**


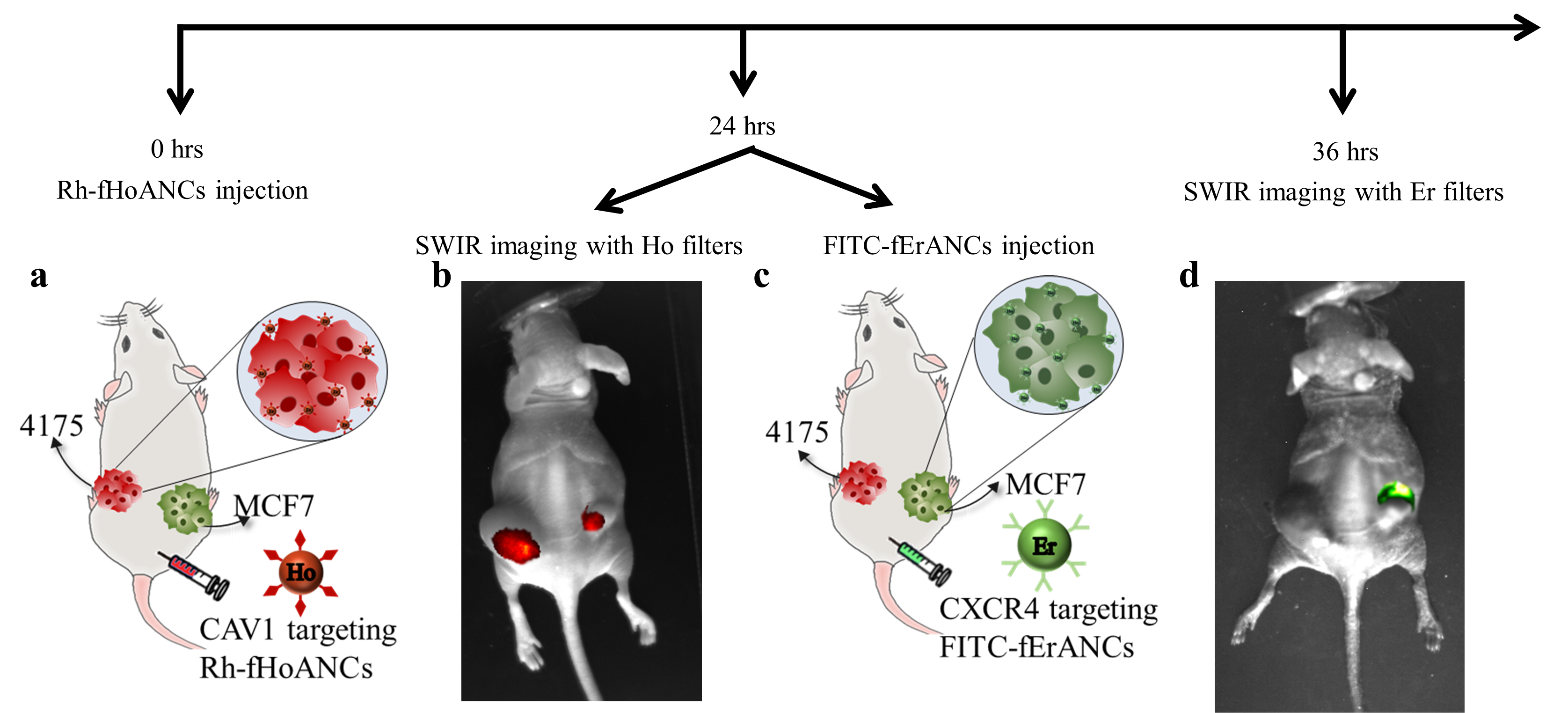


**Supplementary Figure 7: Biomarker specific accumulation of targeted nanoprobes in a single animal following sequential injection:** (a) A bilateral tumor bearing animal was injected with Rh-fHoANCs (targeting 4175 cells). (b) Whole body SWIR imaging showed increased nanoprobe localization in the CAV1-expressing tumor (left flank) compared to the contralateral CXCR4-expressing tumor. (c) The same animal was injected with FITC-fErANCs (targeting MCF7 cells). (d) Whole body SWIR imaging at 36 hrs showed increased nanoprobe localization in the CXCR4-expressing tumor (right flank) compared to the contralateral CAV-1 expressing tumor.

**Supplementary figure 8:**


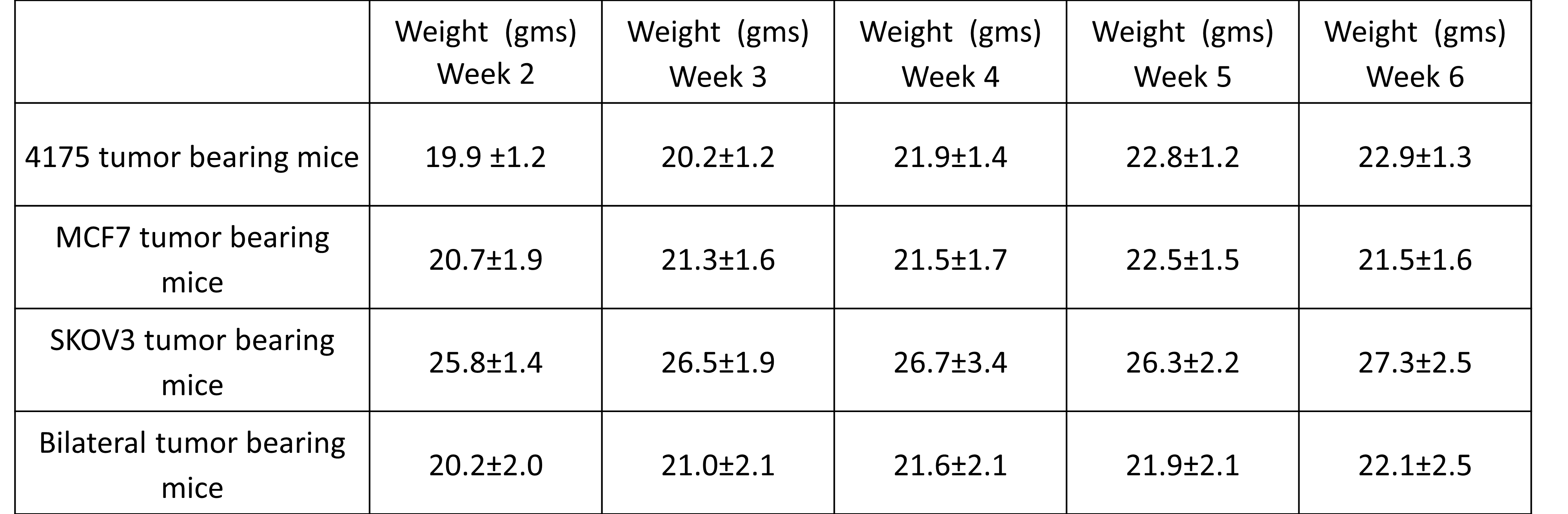


**Supplementary figure 8: Animal weights monitored through study course:** Animal weights per tumor group monitored through the course of the study (from inoculation of tumor cells till sacrifice) are shown in table above. Data is represented in weight (grams); mean ± standard deviation.


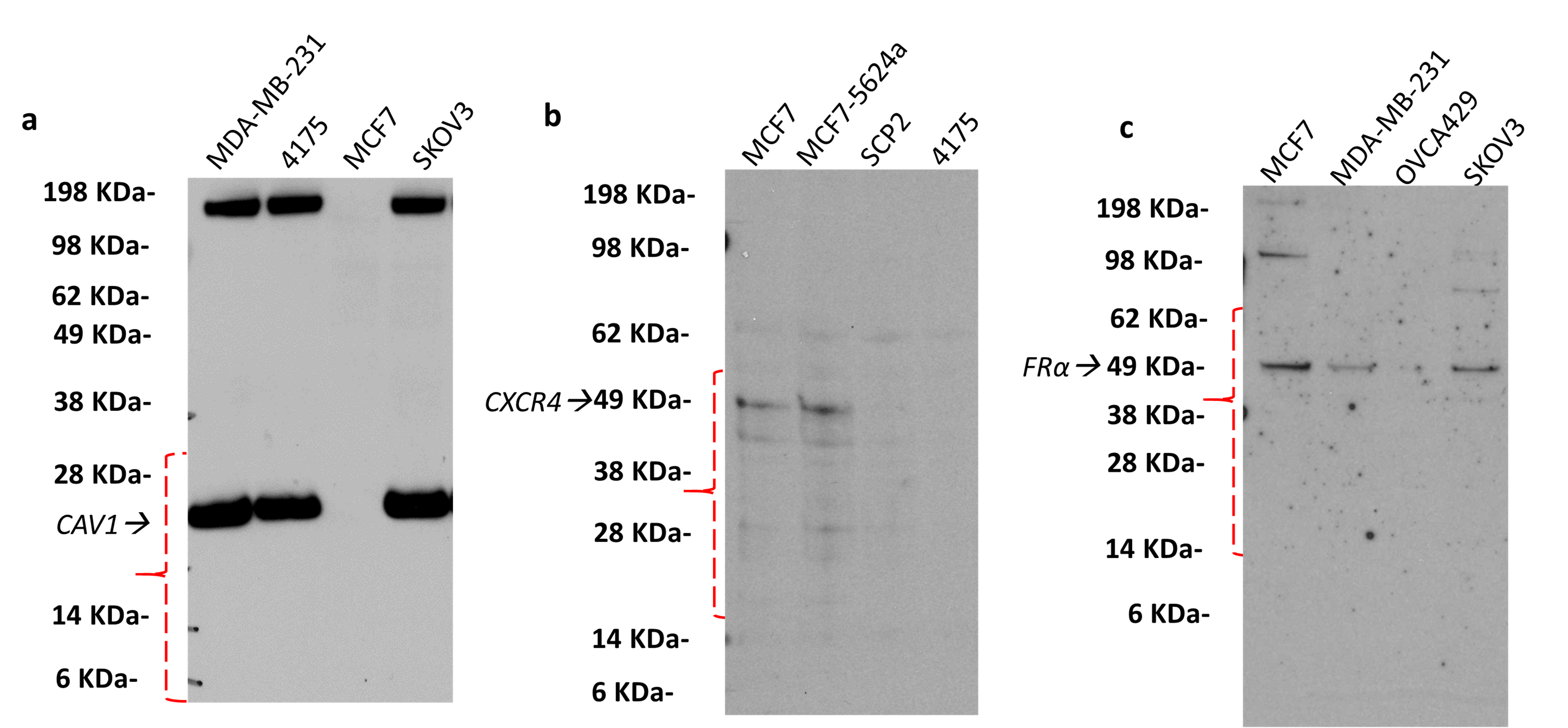
**Supplementary figure 9:**

**Supplementary figure 9 : Full length western blots for Caveolin-1, CXCR4 and Folate receptor alpha proteins -**  The full length western blots corresponding to the cropped western images presented in figure 2 are shown above. The cropped areas of interest represented in figure 2 are indicated by red arrows for each protein. Panel (a) shows the full-length western blot probing for Caveolin-1 presence in triple negative breast cancer line – MBA-MB-231 and 4175, Luminal A breast cancer line – MCF7 and Ovarian cancer cell line of SKOV3. Full length western blot in panel (b) explores the presence of CXCR4 protein in Luminal A breast cancer line – MCF7, MCF-5624a (derived from MCF7 parental cells), SCP2 ( derived from MDA-MB-231 parental lines) and triple negative breast cancer cells of 4175. The folate receptor alpha protein is examined in full-length western blot shown in panel (c) in MCF7(Luminal A breast cancer), MBA-MB-231 (triple negative breast cancer line) and in ovarian cancer cell lines of OVCA429 and SKOV3.
